# On a potential morpho-mechanical link between the gluteus maximus muscle and pelvic floor tissues

**DOI:** 10.1038/s41598-023-50058-8

**Published:** 2023-12-21

**Authors:** Maximilian Siess, Hanno Steinke, Johann Zwirner, Niels Hammer

**Affiliations:** 1 Division of Macroscopic and Clinical Anatomy, Gottfried Schatz Research Center, Medical University of Graz, Auenbruggerplatz 25, 8036 Graz, Austria; 2 Institute of Anatomy, University of Leipzig, Leipzig, Germany; 3 Institute of Legal Medicine, University Medical Center Hamburg-Eppendorf, Hamburg, Germany; 4 Department of Orthopedic and Trauma Surgery, University of Leipzig, Leipzig, Germany; 5 Medical Branch, Fraunhofer Institute for Machine Tools and Forming Technology (IWU), Chemnitz, Germany

**Keywords:** Urology, Urogenital diseases

## Abstract

Stress urinary incontinence presents a condition not only found in female elderlies, but also in young athletes participating in high-impact sports such as volleyball or trampolining. Repeated jumps appear to be a predisposing factor. Yet the pathophysiology remains incompletely elucidated to date; especially with regard to the influence of the surrounding buttock tissues including gluteus maximus. The present study assessed the morpho-mechanical link between gluteus maximus and the pelvic floor female bodies. 25 pelves obtained from Thiel embalmed females were studied in a supine position. Strands of tissues connecting gluteus maximus with the pelvic floor obtained from 20 sides were assessed mechanically. Plastinates were evaluated to verify the dissection findings. In total, 49 hemipelves were included for data acquisition. The fascia of gluteus maximus yielded connections to the subcutaneous tissues, the fascia of the external anal sphincter and that of obturator internus and to the fascia of the urogenital diaphragm. The connection between gluteus maximus and the urogenital diaphragm withstood an average force of 23.6 ± 17.3 N. Cramér φ analyses demonstrated that the connections of the fasciae connecting gluteus maximus with its surroundings were consistent in the horizontal and sagittal planes, respectively. In conclusion, gluteus maximus is morphologically densely linked to the pelvic floor via strands of connective tissues investing the adjacent muscles. Though gluteus maximus has also been reported to facilitate urinary continence, the here presented morpho-mechanical link suggests that it may also have the potential to contribute to urinary stress incontinence. Future research combining clinical imaging with *in-situ* testing may help substantiate the potential influence from a clinical perspective.

## Introduction

Stress urinary incontinence (SUI) is defined as an involuntary loss of urine on effort or physical exertion^1^. Urinary incontinence presents a condition primarily found in female elderlies. Recent studies also suggest a high prevalence of SUI in young, nulliparous, female high-performance athletes. Female athletes involved in high-impact sports such as volleyball or trampolining have been reported to have the highest prevalence, as repeated jumps seem to be a provoking factor in the onset of SUI^2,3^.

The underlying pathophysiology of urinary incontinence remains incompletely elucidated to date. Petros and Ulmsten^4^ stated that both stress and urge incontinence might be caused by a laxity of the vaginal tissues, either from a weakened vaginal wall or from the connective tissues, investing muscles and ligaments. DeLancey proposed the hypothesis that the female urethra is compressed against a continuity of the endopelvic fascia, vaginal wall, arcus tendineus fasciae pelvis as well as the levator ani during the filling of the bladder, labelling it the “hammock hypothesis”^5^. Laxity of the aforementioned structures could thus contribute to the onset of SUI^5^. The pathophysiology of SUI is considered multifactorial, partly evolving as a consequence of weakened supporting structures.

Further to the hypotheses on weakened supportive structures of the pelvis, it remains unclear to date if the surrounding buttock tissues including muscles such as gluteus maximus would partially influence urinary continence by exerting a lateral muscle force directed to the pelvic floor. Recent studies examined the effect of pelvic floor training alone compared to pelvic floor training combined with strengthening of hip synergistic muscles including the gluteus medius, maximus and hip adductor muscles^6^. For the latter combined training, a significant improvement was found in urine leakage compared to pelvic floor training alone^6^. Another recent study showed a co-activation of the pelvic floor and gluteus maximus when walking and jogging^7^. A peak activation was observed during single-leg support activity, however, without complete co-activation^7^. Soljanik et al.^8^ in their study examined the connection of gluteus maximus to levator ani (LA) using functional magnetic resonance imaging and surface electromyography. Changes were observed in the surface area of the ischioanal fossa and gluteus maximus and levator ani, suggesting their synergistic function^8^.

Gluteus maximus appears to functionally impact SUI. Vice versa, it may also provide support for urinary continence. The potential morphological interaction for this supporting mechanism, however, remains incompletely elucidated to date. Potential morphological connections may reach through the ischioanal fossa. De Blok evaluated the connective tissue connections in the ischioanal fossa dissecting eight female pelves combined with plastination and histological analyses. He described a network of septa-like fibers including connections of gluteus maximus, the obturator internus and levator ani^9–11^. In addition, De Blok observed different configurations of these septa depending on the filling of the rectum, suggesting an assisting function in increasing the diameter of the viscera^10^.

To our best knowledge, the morpho-mechanical connections of gluteus maximus and the pelvic floor has not been examined at this point. Hence, information about the basic morpho-mechanical constitution is needed, especially in regard to a potential influence on stress urinary incontinence. It was hypothesized that gluteus maximus could exert a lateral traction force onto the sphincter muscles embedded in the pelvic floor respectively the urogenital diaphragm via connective tissue strands. Hereby, the gluteus maximus might alter the contraction ability of the urogenital diaphragm, hence facilitating the development of SUI.

This given study aimed to examine a potential morphological link between gluteus maximus and the pelvic floor muscles in Thiel-embalmed pelves assessing tissue connections on a macroscopic and mesoscopic level. A second goal was to conduct preliminary mechanical testing, if such connections could be illustrated.

## Material and methods

### Study population

Pelves of 27 female bodies embalmed using the modified Thiel technique^12–14^ were dissected. This specific type of Thiel embalming offers one of the standard techniques in Graz. It has been chosen as it maintains more realistic haptic and optic tissue properties than formaldehyde or ethanol-based techniques^12^. As part of a preliminary trial, two female pelves were dissected for trialing purposes. These bodies were injected with an arterial mass containing dextrin, latex and tetroxide^15^ to better visualize the neurovascular structures within the connective tissues. Age at death averaged 84 ± 10 years (minimum 57 years, maximum 86 years). Twenty tissue samples obtained from ten bodies were used for biomechanical testing (mean age 79 ± 12, minimum 57, maximum 94 years). Further thin slice plastinates were used from a 76-year and an 88-year-old female body. All body donors while alive had given their informed consent to the donation of their post mortem tissues for research. Inclusion criteria for this study were female sex and an intact pelvic floor as well as fully intact hip muscles. Exclusion criteria comprised any known pre-existing alterations at the pelvic floor including trauma or surgery in the perineal area, neoplasms in the pelvic floor, ischioanal fossa or the anal canal region. Approval was granted by the Ethics Committee of the Medical University of Graz (protocol number 32–377 ex 19/20). All methods were performed in accordance with the relevant guidelines and regulations.

### Anatomical approach to the ischioanal fossa

All dissections took place with the bodies placed in a supine position. Two dissections were considered explorative to define the best possible approach to the ischioanal fossa, while preserving the in-situ topography as best as possible. These two specimens were excluded from further data acquisition. Literature was used to refine the surgical approach to the pelvis and pelvic floor^9^. However, the given approach differs, as the dissection was continued to the ventral portion of the ischioanal fossa and the pelvic floor was also approached from caudally. All dissections were carried out by the first author with consistent feedback from one of the senior group members.

**Figure 1:**
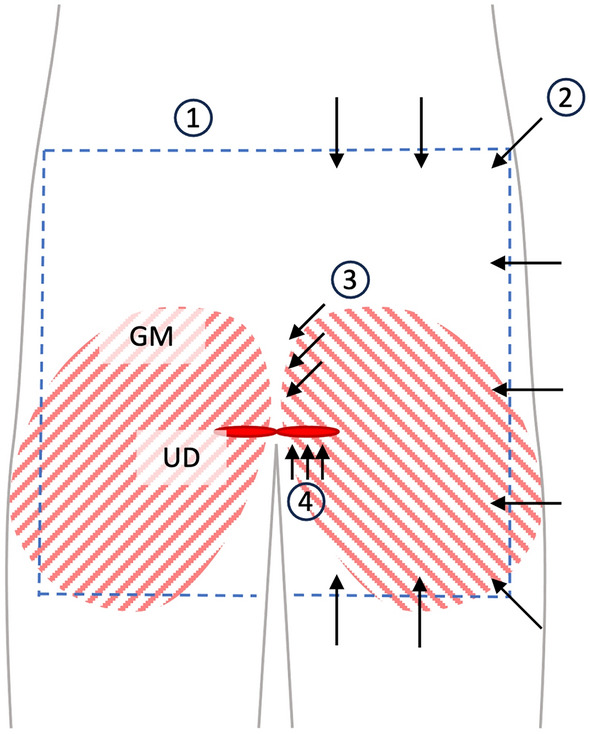
Schematic representation of the anatomical approach to the ischioanal fossa, dorsal view: 1: The blue dotted line marks the course of the skin incision; 2: The black arrows show the direction of preparation of the gluteal and thigh fascia; 3: Blunt opening of the ischioanal fossa in course of the arrows; 4: Preparation of the urogenital diaphragm from caudally. GM—gluteus maximus, UD—urogenital diaphragm.

The dissection approach comprised the following four steps (Fig. 1):

1. *Skin incision* The iliac crest served as a landmark for determining the level at which the horizontal incision was made. This incision was extended to both sides. Then, a longitudinal incision followed bilaterally, beginning at the lateral portion of the iliac crest and extending approximately to the central aspect of the femur. A second pair of horizontal incisions was made at the middle of the thigh.
2. *Anatomical dissection of the gluteal and thigh fascia* The fascia of gluteus maximus was identified at the craniolateral border of the skin flap. Dissection was continued at this corner, thus extending to the medial border of gluteus maximus. Adhesions between the subcutaneous tissues and the muscle fascia were removed sharply. Surgical opening was interrupted once the ischial tuberosity was reached, and dissection of the hamstring fascia began. This step concluded once the origin site of the hamstrings became visible at the ischial tuberosity.
3. The *ischioanal fossa* was exposed bluntly, beginning at the cranial aspect in proximity to the sacrum. Collagen-rich connective tissues were gently divided from adipose tissue by applying soft pressure exerted with the tip of the index finger. The connections between gluteus maximus fascia and the subcutaneous tissues were dissected sharply at the medial border of the muscle fascia to enhance visibility. This step allowed identifying the following structures inside the ischioanal fossa: obturator internus, levator ani, external anal sphincter, urogenital diaphragm, pudendal canal, inferior rectal vessels and rectal nerve.
4. Visualization of the *urogenital diaphragm* began once the inferior ramus of the ischium was palpated. It served as a landmark for the remaining dissection. First, the urogenital diaphragm was prepared from caudally. Connective tissues between the inferior ramus of the ischium and skin of the inner thigh were dissected in layers from dorsal-caudal to ventral-cranial. These steps allowed ensuring the structural integrity of the urogenital diaphragm. The resulting connective tissue flap was removed at the thigh once ischiocavernosus and the urogenital diaphragm became visible. A careful dissection of the dense connectives of the gluteal fascia and subcutaneous tissues followed at the region of the ischial tuberosity. This substep concluded once the inferior border of the gluteus maximus was reached. Throughout step four, the ischioanal fossa was cleared of redundant connective and region fatty tissue. Following this approach allowed to assess the regional anatomy of the ischioanal fossa. Further, this exposure allowed collecting morphological findings in line with the pilot trials.

### Data collection

To ensure reproducibility and a high level of standardization in data acquisition, the following three transverse and three sagittal planes were assessed.

**Table:** 

Level	Transverse plane	Sagittal plane
1st	Skin	Medial border of the ischial tuberosity/obturator internus
2nd	Pudendal canal/upper border of the obturator internus	Ischioanal fossa
3rd	Deep ischioanal fossa/cranial to the pudendal canal	Pubic symphysis/pelvic diaphragm/urogenital diaphragm

To gain a general understanding of the possible connections of the gluteus maximus to the pelvic floor, parameters were collected using a case report form which included the following details:

- *Connections*: Which muscles/connectives tissues were found within the given plane?
- *Arrangement:* Were such connections present unilaterally or bilaterally?
- *Symmetry*: Symmetrical versus asymmetrical. Assessment was conducted while pulling the skin flap contralaterally until tension of the urogenital diaphragm was achieved.
- *Angle of insertion*: sharp, flat or perpendicular angle. Assessment as above.
- *Quantity of fibers:* referring to the origin at the lateral border of the ischioanal fossa.
- *Composition*: Were the given connections composed of connective tissue, neurovascular bundles, or muscle tissue?

### Plastination

The experimental data was verified using the Leipzig plastinate collection. In brief, fresh frozen blocks were sectioned, freeze substituted in acetone, and vacuum embedded to epoxy resin^16^. Recently, selected plastinates were stained again using the PAS method to distinguish different collagen types, which give different staining^17^.

### Biomechanical testing

**Figure 2:**
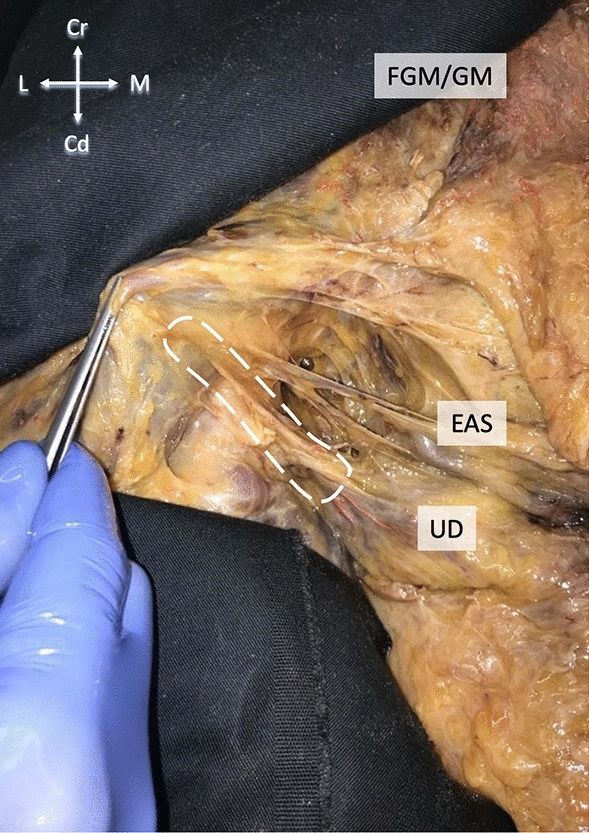
Ischioanal fossa on the left side of a body donor in a supine position. The gluteus maximus is lifted dorsally. The white dashed line represents the approximate course of sample retrieval for the biomechanical testing. After retrieval, the tissue was size adjusted to fit the length of the clamping (40 mm). EAS—external anal sphincter, FGM—fascia of gluteus maximus, UD—urogenital diaphragm.

Samples from 20 sides (10 individuals) were used for biomechanical testing until material failure. The samples were retrieved sharply including about 15 mm of gluteus maximus and about 15 mm of the urogenital diaphragm (Fig. 2). Sample retrieval began with the resection of gluteus maximus. The incision was extended along the inferior ramus of the ischium close to the ischiocavernosus, with the bone contact adhered to minimize damage to the structures of interest. To bridge the time between sample gathering and testing, the specimens were then stored in a Thiel container solution^18^. Prior to the biomechanical trials, the tissues were size (length and width) adjusted for standardization purposes and to exclude testing of muscle tissue. The final length of the sample was reduced to 40 mm with a clamp-to-clamp distance of 20 mm and a maximum width of 10 mm. A Z020 materials testing machine was used for uniaxial load-to-failure testing, equipped with an Xforce P load cell of 2.5 kN (ISO 7500 accuracy grade 1) and pneumatic clamps (all ZwickRoell AG, Ulm, Germany). The samples were processed and clamped using 3D-printed clamps as stated elsewhere^19–21^. Customized clamps with sharp pyramids were used to minimize material slippage^19,22^. This highly standardize experimental setup has previously rendered offering reliable and accurate material properties for various tissues involving the layered human neurocranium, pelvis and tendon tissues^20,21,23–31^. All samples were preconditioned with five load-unload cycles of up to 1 N before testing until material failure. A displacement rate of 0.1 mm/s was used.

### Statistical evaluation

The data were analyzed using Prism version 9 (GraphPad Software Inc., La Jolla, CA, USA), SPSS version 28 (IBM, Armonk, VA, USA) and Microsoft Excel version 16.49 (Microsoft Corp., Armonk, NY, USA). Following the testing for normal distribution, the Chi-squared test, Fisher’s exact test and Cramér’s φ correlations were determined to compare categorial items between symmetry and connections. Values of Φ between 0.1–0.3, 0.3–0.5 and ≥ 0.5 were considered moderate, strong and very strong, respectively^32^. *P* values ≤ 0.05 were considered statistically significant.

## Results

49 hemipelves from 25 bodies were studied for data inclusion. One hemipelvis was excluded due to the formation of scar tissue (potentially as sequalae of abscess formation) affecting the topography at the transition zone between gluteus maximus and the ischioanal fossa. The ischioanal fossa was completely taken up by the scar tissue which made assessing the various potential connections for this hemipelvis unfeasible. The dissections yielded a series of connective tissue connections originating from gluteus maximus, attaching it to obturator internus, levator ani and the urogenital diaphragm. These connections could further be verified using the plastinated body slices.

### Gluteus maximus is consistently connected to the ischioanal fossa via fibrous connections but also via fascial continuations

**Figure 3:**
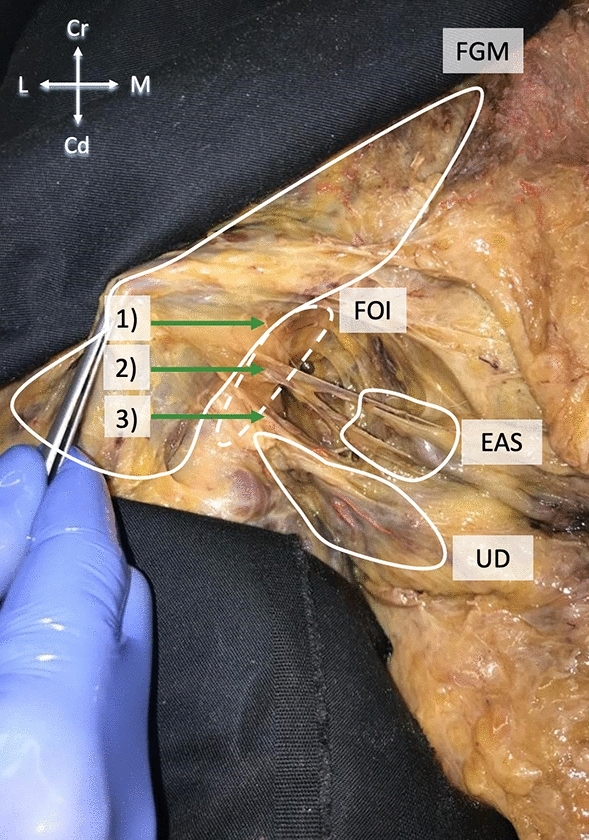
Ischioanal fossa on the left side of a body donor in a supine position. The gluteus maximus is lifted dorsally. Number “(1)” depicts the connection of FGM to FOI. The link of FGM to SAE, comprising the inferior rectal vessels and rectal nerve, is labeled as “(2)”. Marked “(3)” is the connective tissue originating from FGM and investing in the urogenital diaphragm. This connection is used for the biomechanical testing in the given study. EAS—external anal sphincter, FGM—fascia of gluteus maximus, FOI—fascia of obturator internus, UD—urogenital diaphragm.

At the posteromedial border and in the area of the entrance to the ischioanal fossa, the fascia of gluteus maximus yielded attachments to the subcutis via multiple small septa-like fascia extensions. These extensions invested lobules of adipose tissue and were similarly structured as the adipose tissues found in the ischioanal fossa (Fig. 3). In all of the 49 included hemipelves this connection was found bilaterally (100%), and symmetrical in 21 cases (88%). At the skin level, no muscle fibers nor neurovascular bundles were observed macroscopically for the connections between gluteus maximus and subcutis.

A second connection of the fascia of the gluteus maximus was found at its medial border. The cranial two-third overlay the fascia of the obturator internus in an approximately sagittal plane. The fascia of gluteus maximus covered the inferior ramus of the ischial bone and folded ventrally to continue into the fascia of the obturator internus, providing a dorsal-lateral border for the ischioanal fossa. These fasciae were in contact in all cases (100%). At its medial border, the fascia of gluteus maximus was connected to the subcutis dorsally, overlying the fascia of obturator internus ventrally.

**Figure 4:**
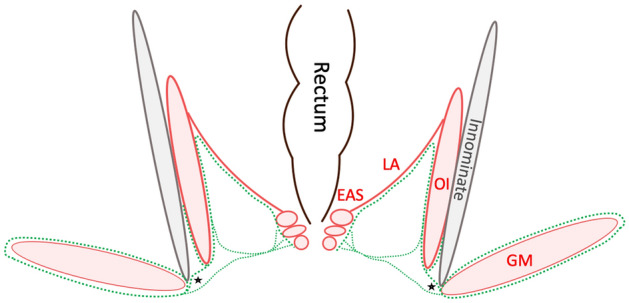
Schematic overview of the fasciae (green dotted lines) in the ischioanal fossa in a coronal section. The asterisks mark the connection of FGM and FOI and, secondly, the relationship of EAS and FGM. The fasciae sheath the inferior rectal vessels and rectal nerve. EAS—external anal sphincter, GM—gluteus maximus, OI—obturator internus, LA—levator ani.

The ischioanal fossa at this level was filled with a mixture of fibrous and adipose connective tissues, forming a three-dimensional entity that interconnected the borders of the fossa. The inferior rectal vessels and rectal nerve traversed through this network of fibers and fatty tissue following a transverse plane. The neurovascular bundles penetrated the pudendal canal laterally. Here, the fascial connections of gluteus maximus and obturator internus provided a sheathing for the neurovascular pathways to the external anal sphincter and the skin (24/24, 100%) (Fig. 4). In 71%, these fibers appeared symmetrical (17/24). In 29% (7/24), they were found to be asymmetrical. The connection comprised one fiber strand in 98% of the hemipelves (48/49) and two strands in one case (2%, 1/49 hemipelves). The connections mostly inserted at a sharp angle (46/49, 93.8%), less frequently at a perpendicular angle (3/49, 6.1%).

**Figure 5:**
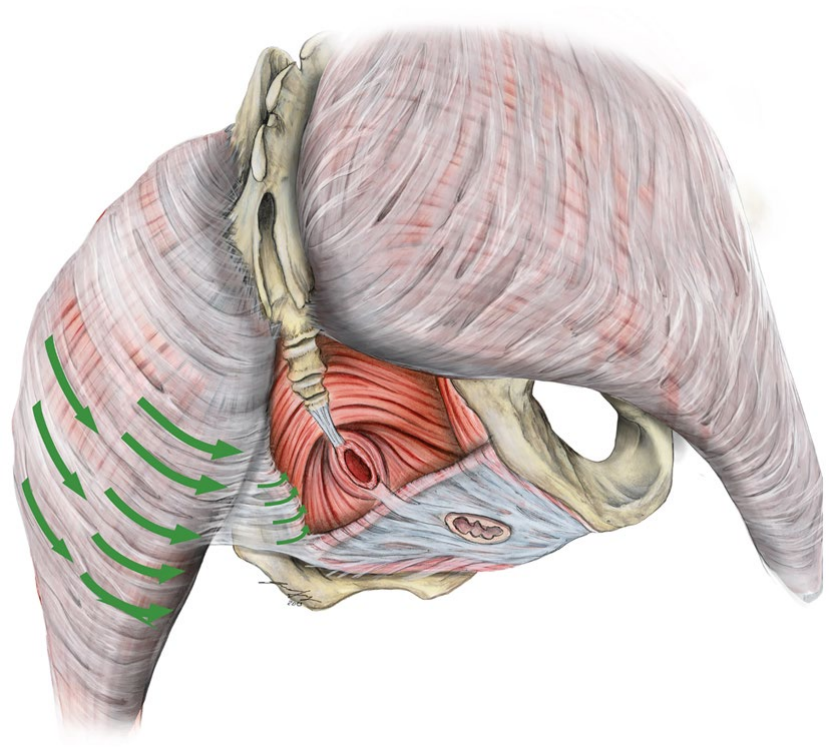
Overview of the topography of the pelvic floor and the gluteus maximus. Green arrows mark the course of fascial continuations alongside the gluteus maximus, obturator internus and urogenital diaphragm.

**Figure 6:**
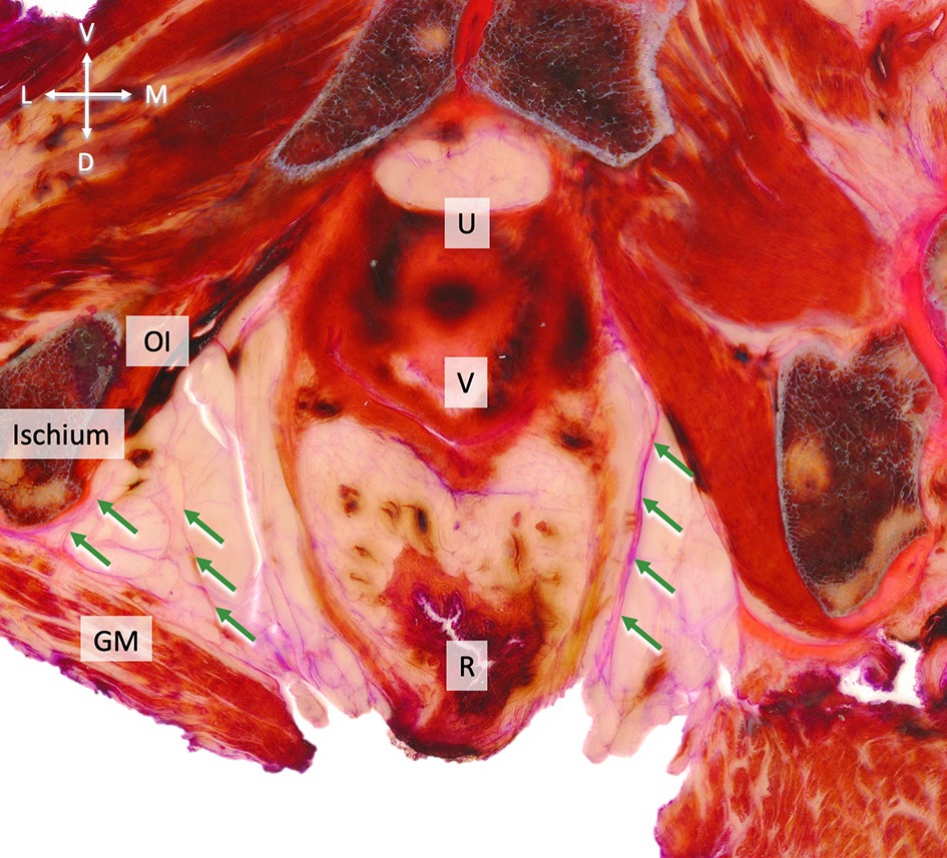
Transverse view of the ischioanal fossa in a thin slice plastinate stained with PAS of a 88-year-old female body. Type III collagen fibers are stained in pink, Type I collagen fibers mostly in dark red. The arrows mark the connective tissue fibers inside the ischioanal fossa connecting adjacent muscles including the gluteus maximus, levator ani, obturator internus and the urogenital diaphragm. The ischioanal fossa is void of muscle fibers. A lamella reaches from the OI dorsally, and from this fascia other zigzag-aligned fibers towards gluteus maximus. GM—gluteus maximus, OI—obturator internus, LA—levator ani, R—rectum, U—urethra, UD—urogenital diaphragm, V—vagina.

**Figure 7:**
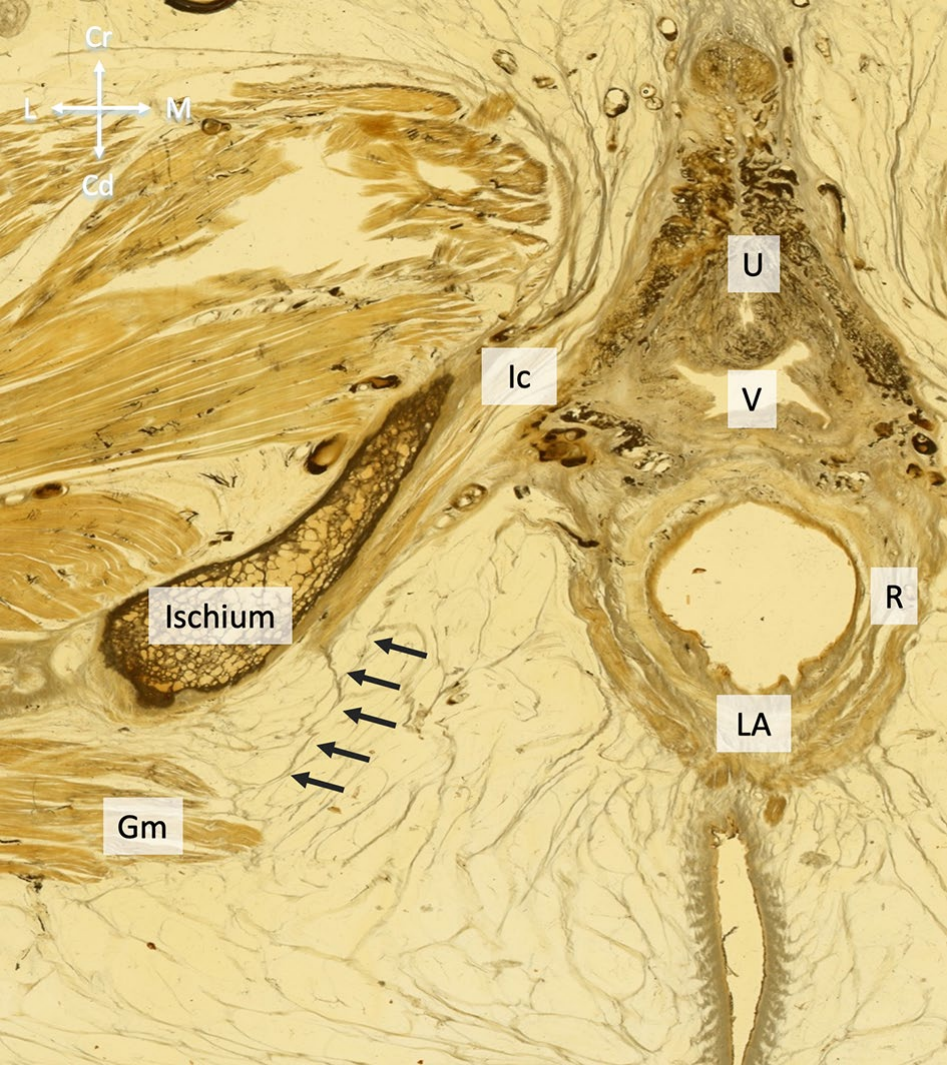
Transverse thin slice plastinate of a 76-year-old female body donor. The arrows mark the connective tissue fibers originating from the FGM and connecting it to ischiocavernosus, respectively, the urogenital diaphragm. FGM—fascia of gluteus maximus, LA—levator ani, Ic—ischiocavernosus, Is—Ischium, R—rectum, V—vagina, U—urethra.

More laterally, the fascia of gluteus maximus covered the inner surface of the ischial tuberosity in contact with bone. It hereby thickened, yielding a skin-like appearance. Now following the pudendal vessels and nerves within the pudendal canal, the fasciae connecting gluteus maximus and obturator internus extended more inferiorly to the urogenital diaphragm investing neurovascular branches to the urogenital diaphragm and the perineal region (Figs. 3, 5). No muscle fibers could be identified at this area (49 hemipelves, 2 plastinate series, 100%). Eventually, the branches reached their area of supply laterally in an almost horizontal plane. The connection was present bilaterally in all cases (24/24, 100%), symmetrical in 18 cases (75%) and asymmetrical in six cases (25%). In Figs. 6 and 7, the connections of gluteus maximus and the adjacent muscles, traversing through the fat tissue in the ischioanal fossa, are illustrated in thin slice plastinates. Gluteus maximus connections were consistent among the levels and varied in tensile strength but consistently represented mechanical properties of dense connective tissues (Figs. 3, 5).

Cramér φ analyses demonstrated that the connections of the fasciae between gluteus maximus were consistently strong in all horizontal planes (φ ≥ 0.73, *p* ≤ 0.020), in all sagittal planes (φ = 1.00, *p* ≤ 0.040), and between the horizontal and sagittal (φ ≥ 0.77, *p* ≤ 0.037) planes, respectively.

**Figure 8:**
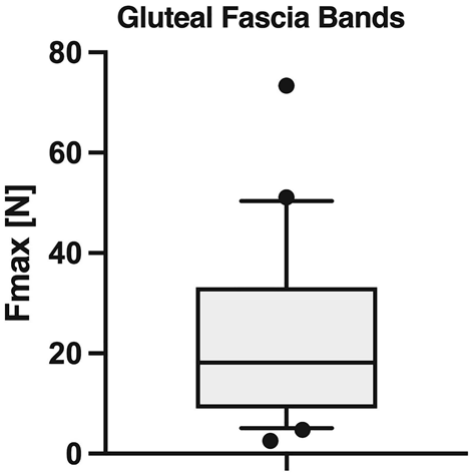
Boxplot diagram displaying the maximum force (F_max_) the connective tissues withstood under strain. Outlines of the boxes indicate the 25th and 75th percentile, the solid horizontal line the median. Whiskers represent the minima and maxima. On average, F_max_ averaged 23.6 ± 17.3 N (range 2.6–73.4 N).

The samples used for the biomechanical testing comprised the connection of the fascia of gluteus maximus and obturator internus reaching the urogenital diaphragm in a mostly sagittal plane from dorsally. This tissue presents a direct connection from gluteus maximus to the urogenital diaphragm, hence, potentially distributing load. The maximum force the tissues withstood under strain averaged 23.6 ± 17.3 N (range 2.6–73.4 N; Fig. 8).

## Discussion

The present study aimed at investigating morpho-mechanical connections of the gluteus maximus to the pelvic floor macroscopically. If such connections could be illustrated, a second goal was to examine these connections morphologically in detail and assess their loading capacity on a subset of samples. The gluteus maximus and its investing fascia were shown to be connected to the obturator internus and the urogenital diaphragm via fascial continuations. This fascia then extends and connects to the fascia of levator ani. Sheathing the inferior rectal nerve and inferior rectal vessels, the fasciae of gluteus maximus and obturator internus are also connected to the external anal sphincter. Throughout the dissections, no muscle fibers were found within these connecting bridges.

### Dense connections appear to exist between gluteus maximus, obturator internus and the urogenital diaphragm

It was found that the sheathing of the inferior rectal vessels and the inferior rectal nerve not only comprised “extensions” of the fascia of the obturator internus, but also receives fiber-strands from the fascia of gluteus maximus. Kurihara et al.^33^ in their study described the connection between the pudendal canal of Alcock and the anal canal^33^. These researchers named this connection the “septum of the ischiorectal fossa” (SIF), which separates the ischioanal fossa into an “inferior levator space” and a “clinical ischiorectal space”. However, it remained undescribed if the origin of the septum of the ischiorectal fossa also comprised parts of the fascia of gluteus maximus or just the fascia of obturator internus^33^. In the present study, the SIF comprised fiber strands originating from the fascia of gluteus maximus and obturator internus. One case report illustrated a sigmoid-gluteal abscess with a fistula traversing through the ischioanal fossa and the obturator internus^34^, providing indirect evidence in favor of such connection. Therefore, the question arose if the septum of the ischiorectal fossa and its extensions towards the gluteal fascia might pose a pathway for abscess formation extending to the gluteal region. The continuity of the fascia of gluteus maximus into the obturator internus fascia was previously demonstrated by other authors: Zhang et al.^35^ described the morphology of the ischioanal fossa in males, including a “dense strip-fiber connecting with junction fascia between the obturator internus and gluteus maximus”. These fibers continue into the fasciae of the obturator internus, gluteus maximus and levator ani. Such detailed description is pending for females to date. De Blok described “half-circular connective tissue ribbons" connecting obturator internus, levator ani and gluteus maximus^11^. Towards the center of the perineum, these structures have been described as being aligned sagittal and laterally the fibers being aligned transversely. Medial fibers were attached to the superficial perineal fascia, whereas the lateral fibers had attachments to the fascia of gluteus maximus, obturator internus and the ischial tuberosity. This pattern goes in line with the data acquired in the present study. Here, the fascia of gluteus maximus and obturator internus reached the urogenital diaphragm in a mostly sagittal plane coming from dorsally. This connection can be seen as a continuation of the fascia. DeBlok describes that in between the aforementioned septa, tunnel-like structures appear to exist^9^. These findings were confirmed in females in the given experiments. However, as the blunt preparation extended into the deeper parts of the fossa and as the adipose connectives were removed, these fibers were mostly gone. These findings indicate that the collagen connectives of this region help keep fatty tissue in place, and to lesser extent transmit force to the pelvic floor. Macroscopically, no muscle fibers were present in the connective tissues of the ischioanal fossa. However, Thiel-embalming might have dissolved muscle fibers potentially located in the fossa^21^. In contrast to our findings presented here, De Blok found that the given septa comprised connective tissue fibers, fibroblasts, small blood vessels and smooth muscle cells^11^. Using serial sections of female pelves, De Blok also found that the connective tissue in the ischioanal fossa forms an entity continuing into the fasciae of the pelvic muscles and the above-mentioned muscles^10^. The septa formed by the connectives had medial extensions to the labia majora and the fascia perinei superficialis^10^. On the lateral border of the fossa, at the region of the ischial tuberosity, a thickening of the covering fascia was observed, yielding a skin-like texture.

The consistency of the given fasciae connections and symmetry of gluteus maximus in the horizontal and sagittal planes has been demonstrated for females in the given experiments by the Cramér φ analyses and Fisher’s exact tests. These statistical considerations are strongly indicative of gluteus maximus fulfilling a consistent role in contributing to pelvic floor morpho-mechanics.

Steinke describes this as being an extension of the gluteal suspension system^36^. The gluteal suspension system originates from the ischial tuberosity and is connected to the iliotibial tract, the fascia of gluteus maximus, the aponeurosis of the gluteal muscles, and the fascia lata. Steinke describes based on morphological findings that the gluteal suspension system is involved in forming the gluteal sulcus and reaches to the ischioanal fossa covering the inferior border of the gluteus maximus^36^. The thickening of gluteal fascia over the ischial tuberosity, which was observed in the present study, might be caused by lateral movement of the muscle when sitting down and, thereby, exposing the underlying fascia^37^.

### Functional mechanical evidence in favor of the morpho-mechanical link of gluteus maximus and the urogenital diaphragm

While the main focus of the given study was set on the morphological connections of gluteus maximus and the pelvic floor, Soljanik et al.^8^ made attempt to evaluate the role of the ischioanal fossa in the context of functional interaction. They examined gluteus maximus and levator ani activity during voluntary pelvic floor contraction, using surface electromyography and functional imaging. Soljanik et al.^8^ found a synchronous movement in the same direction of the levator ani, gluteus maximus, and the ischioanal fossa. Taken together, these results are indicative of the functional morpho-mechanical connection of gluteus maximus and the pelvic floor.

The maximum force the connective tissues within the fascia of gluteus maximus and the urogenital diaphragm withstood, appear similar to connective tissues in other body regions with load bearing capacity such as dura mater^27^. This feature suggests that the function of the connective tissues might be an “in-place-keeping” and structuring of the adipose tissue and, to some extent, force transmission to the pelvic floor. However, as the connective and fatty tissue form a unit in the ischioanal fossa, a conclusion on the overall biomechanical function of this unit cannot be drawn since only fiber strands located on the lateral border of the fossa were tested.

The here presented morpho-mechanical findings give first preliminary insights into the potential connection of gluteus maximus and the urogenital diaphragm forming part of the pelvic floor. Excessive traction exerted via gluteus maximus may facilitate lateral traction to the sphincters embedded in the pelvic floor, thereby resulting in urinary incontinence. Though gluteus maximus has also been found to be a muscle aiding in urinary continence when trained adequately, unfavorable loading in single leg stance may undermine this function towards pathology.

### Limitations

The study population comprised donors with a limited age range of 57–86 years. A younger study population would have been desirable to extrapolate the findings on pelvic floor dysfunction on the cohort of young female athletes more precisely, removing the bias of aging and postmenopausal effects. This bias does also account for the biomechanical analysis. Age has previously been shown as one of the factors with influence on the load-bearing characteristics of human musculoskeletal tissues^38,39^. Further to this, a larger sample number would have been desirable to present more accurate material data^40^. Combined imaging and functional testing would be another prospect to account for in future trials. Furthermore, the dissection of embalmed tissues poses another limitation. Anatomy and topography could not be preserved in their entire structural integrity, even though the preparations were conducted carefully.

As stated elsewhere^18,21,41,42^, Thiel embalming influences biomechanical properties to an extent so that its use should be limited to preliminary mechanical testing^21^. In comparison to testing of fresh-frozen tendons, mechanical trials of Thiel embalmed tendons resulted in altered failure characteristics and lower failure stress, indicating a different collagen fiber/network constitution^42^. It was hypothesized that the high contents of boric acid in Thiel embalming may facilitate collagen denaturing which softens the tissues^42^. This suggests that the mechanical results in the present study display biased values of the true loadbearing capacity of region. The removal of fat caused by the Thiel embalming causes another methodological limitation^41^. As a consequence of the Thiel-embalming, and due to the broad variation in both structure and mechanical characteristics among the harvested tissues, the conducted testing can only be considered preliminary. If the specimens of the given experiments are indeed softer than non-embalmed tissue, as reported in the literature^42^, the connection of gluteus maximus to the urogenital diaphragm could potentially have a higher load bearing capacity in fresh specimens. Therefore, to obtain accurate mechanical insights about this connection, future studies should especially focus on examining fresh or fresh-frozen tissue samples. Furthermore, it is essential to adopt a standardized cross-section area for the testing of these tissues, given the limited body of research assessing the maximum force.

Additionally, as the connective and fatty tissue in the ischioanal fossa form a three-dimensional network, uniaxial testing of samples most likely does not reflect the in-situ properties to full extent.

## Conclusion

Gluteus maximus is morphologically linked to the pelvic floor via strands of connective tissues investing the adjacent muscles. This morpho-mechanical link suggests that under certain conditions, gluteus maximus may have the potential to contribute to urinary stress incontinence pathophysiology. On the other hand, it may offer a protective effect on continence^6^. Hence, future research combining clinical imaging with *in-situ* testing such as electromyography may help substantiate the influence of gluteus maximus from a clinical perspective and may aid in providing sufficient training recommendations.

## Data Availability

The data obtained and analyzed during the current study is available from the corresponding author on reasonable request.
